# Cyclooxygenase-2 inhibitor celecoxib attenuates joint contracture following immobilization in rat knees

**DOI:** 10.1186/s12891-016-1303-5

**Published:** 2016-10-24

**Authors:** Junya Ozawa, Akinori Kaneguchi, Ryo Tanaka, Nobuhiro Kito, Hideki Moriyama

**Affiliations:** 1 Department of Rehabilitation, Faculty of Rehabilitation, Hiroshima International University, Kurose- Gakuendai 555-36, Higashi-Hiroshima, Hiroshima 739-2695 Japan; 2 Graduate School of Medical Technology and Health Welfare Sciences, Hiroshima International University, Higashi-Hiroshima, Hiroshima Japan; 3 Department of Rehabilitation Science, Graduate School of Health Sciences, Kobe University, Tomogaoka 7-10-2, Suma-ku, Kobe, Hyogo Japan

**Keywords:** Celecoxib, Joint contracture, Immobilization, Knee, Muscle

## Abstract

**Background:**

The aim of this study is to clarify the following two points: First, whether a cyclooxygenase-2 mediated pathway is involved in the formation of immobilization-induced joint contracture and, second, the effectiveness of oral administration of non-steroidal anti-inflammatory drug celecoxib (CBX) for the prevention of myogenic and arthrogenic contracture following immobilization in a rat model.

**Methods:**

Thirty male rats were randomly divided into three groups: immobilization (Im), Im + CBX, and control (*n* = 10 each). External fixation immobilized the right knee joint of Im and Im + CBX groups in flexion for 3 weeks. 50 mg/kg of CBX was administrated daily to the Im + CBX group during this period. The passive range of motion (ROM) of knee joints was measured before and after transection of knee flexor muscles and myogenic and arthrogenic ROM restrictions were calculated. The semitendinosus muscles and knee joints were investigated histologically to elucidate factors responsible for contracture.

**Results:**

Myogenic ROM restrictions were exhibited both in Im and Im + CBX groups (44 ± 5 and 36 ± 8 °, respectively), but restrictions significantly decreased in the Im + CBX group compared to the Im group. Significant reductions of the muscle length ratios (Rt/Lt) and sarcomere number ratios (Rt/Lt) in knee flexor semitendinosus muscle, which are responsible for myogenic contracture, were also seen both in Im group (92 ± 5 and 92 ± 4 %, respectively) and Im + CBX group (97 ± 3 and 97 ± 3 %, respectively), but were inhibited by CBX administration (*P* < 0.05). Im and Im + CBX groups exhibited arthrogenic ROM restrictions with no significant differences (82 ± 3 and 83 ± 5 °, respectively). Posterior synovial length shortening and pathological changes (hemorrhage in joint cavities and capsule edema) in the knee joints were comparable between Im and Im + CBX groups.

**Conclusions:**

Oral administration of celecoxib partially reduced myogenic ROM restriction concomitantly with knee flexor muscle shortening following immobilization. These results imply that inflammation and nociception are involved in myogenic contracture formation independently of joint immobilization, and that CBX is effective in preventing joint contracture following immobilization in rats.

## Background

The development of joint contractures is induced by a variety of events such as immobilization [1–6], joint surgery or trauma [7], muscle weakness, and neurological dysfunction such as paralysis and spasticity [1, 8]. These events accompany joint inactivity and suggest that joint immobilization is essentially a trigger for contracture formation. If movement of the affected joint is permitted, passive movements (e.g., stretching, continuous passive motion, and splinting) are the most common interventions for the treatment and prevention of joint contractures [1]. However, the efficacy of passive movement is still controversial. A systematic review revealed that dynamic splints, which provide prolonged passive stretching, is a safe and effective treatment for contracture in humans and animals [8]. On the other hand, Prabhu et al. [9] reviewed that it is not clear whether passive movements are effective for the treatment and prevention of contractures. Katalinic et al. [10] also concluded that stretching does not have an important effect on joint mobility. These results imply the need for the development of new alternative methods for the treatment of joint contractures.

In a recent study, we found that contracture is generated in arthritic knee joints with only a slight, transient reduction in active joint range of motion (ROM) during locomotion [11, 12]. This result indicates that joint contracture is not necessarily associated with joint immobilization. Interestingly, joint immobilization leads to not only contracture, but also joint inflammatory conditions characterized by synovitis [13] and an upregulation of inflammation-related genes [14]. In addition, arthritis-induced contracture is partially prevented and improved by anti-inflammatory drugs [15–17]. As such, anti-inflammatory treatment may prevent immobilization-induced contracture as well as arthritis-induced contracture. However, investigation into the effects of anti-inflammatory drugs on immobilization-induced joint contracture is very limited. Michelsson et al. [18] reported that administration of corticosteroid methylprednisolone has a preventive effect on joint stiffening, but only in the beginning of the immobilization period (<5–6 weeks). After this, ROM restriction proceeded despite remobilization of the rabbit knees. It was also shown that the administration of ibuprofen, a non-steroidal anti-inflammatory drug (NSAID), does not suppress the formation of joint stiffening in immobilized rabbit knees [19]. However, ROM analysis was only carried out semi-quantitatively, and more importantly, the report did not distinguish between the contributions of muscular and articular factors on contracture formation; the factors responsible for joint contracture are divided into mainly muscular and periarticular structures [4, 20]. Intra-articular injection of high molecular weight hyaluronan, which modulates inflammatory response, could prevent fibrotic changes in immobilized joint capsules in rats [21] and rabbits [22]. However, they did not evaluate myogenic ROM restriction and focused not on muscular but on articular factors in the contribution of contracture. Thus, the effects of anti-inflammatory drugs on myogenic and arthrogenic contracture formation have not been yet concluded.

In this study, we selected celecoxib (SC-58635; 4-[5-(4-methylphenyl)-3-(trifluoromethyl)-1H-pyrazol-1-yl] benzenesulfonamide), a selective cyclooxygenase (COX)-2 inhibitor, as the anti-inflammatory drug. Celecoxib has been widely used to treat pain and inflammation via suppression of prostaglandin E_2_ synthase [23]. The aim of this study is to clarify the following two points: First, whether a COX-2 mediated pathway is involved in the formation of immobilization-induced joint contracture and, second, the effectiveness of NSAID celecoxib for the prevention of myogenic and arthrogenic contracture following immobilization in a rat model.

## Methods

### Animals

Eight-week-old male Wistar rats (30 animals, 199 ± 3 g, Japan SLC, Shizuoka, Japan) were used in this study. The animals were housed in standard cages, given access to food and water ad libitum, and maintained in a thermoneutral environment (22–25 °C) with a 12-h light-dark cycle.

### Joint immobilization

**Fig. 1:**
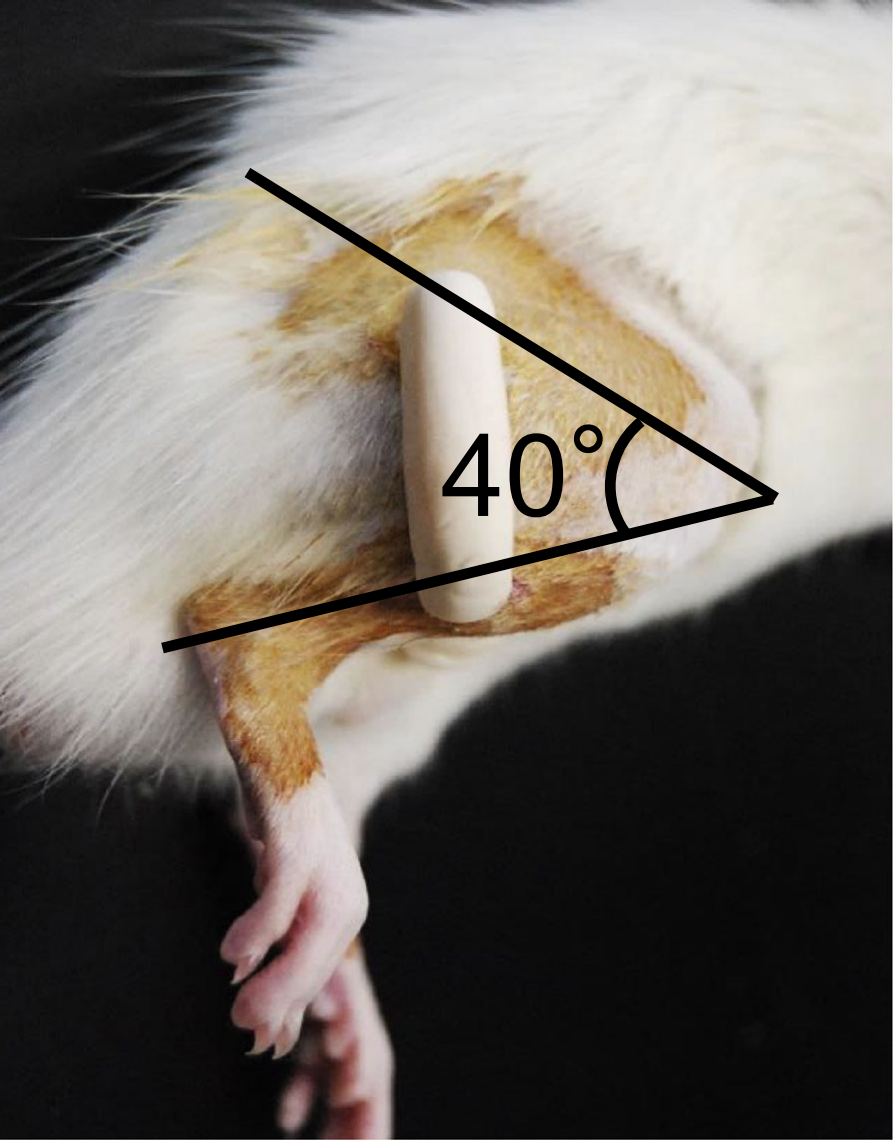
Photograph of joint immobilization. Knee joint is immobilized by an external fixator at approximately 140 ° flexion (the angle between the femur and the tibia is 40 °)

Joint immobilization was conducted using the external fixation method, as described previously [24]. Under anesthesia with an intraperitoneal injection of sodium pentobarbital (0.5 mL/kg), Kirschner wires were screwed into the femur and tibia of the rat’s right hindlimbs and subsequently fixed by wire and resin at 140 ° knee flexion for 3 weeks (Fig. 1). Joint immobilized rats were divided into two groups: immobilization (Im) (*n* = 10) and immobilization with celecoxib administration (Im + CBX) (*n* = 10). Celecoxib (50 mg/kg orally) was administrated daily to the Im + CBX group during the 3 week immobilized period. Age-matched untreated animals were used as the control group (*n* = 10).

### ROM measurement

At the end of the intervention period, passive knee joint ROM was measured as described previously [12]. Rats were anesthetized with sodium pentobarbital (0.5 mL/kg) with supplemental diethyl ether inhalation to inhibit muscle contraction completely, if necessary. After removing an external fixator and exfoliating of skin of the hindlimb, color markers were placed corresponding to the greater trochanter, knee lateral joint space, and lateral malleolus. The femur was fixed at approximately 90 ° hip flexion, and 14.6 N·mm extension moments were applied to the knee joint. Knee joint ROM was measured using a 3D motion analysis system KinemaTracer (KISSEI COMTEC, Nagano, Japan) and three video cameras (Tokina, Tokyo, Japan). The markers in the filmed image were traced. The KinemaTracer software used to analyze the coordinates was calibrated by recording a cube of known size. The angles between the axes of the femur (the greater trochanter to the knee lateral joint space) and fibula (the knee lateral joint space to the lateral malleolus) were measured as the knee extension ROM (ROM+m). After the ROM+m measurement, rats were sacrificed by exsanguination under inhalation of diethyl ether. The knee flexor muscles, except for the semitendinosus muscles, were completely transected at the mid-belly portion. As for the semitendinosus muscle, the ischium tuberosity attached to the tendon was removed from the hip bone. After excluding myogenic factors in flexion contracture, knee extension ROM (ROM-m) was measured again as detailed above. All measurements were performed on both sides of the knee in all groups.

Myogenic ROM restriction was defined as contracture due to knee flexor muscle components, and arthrogenic ROM restriction was defined as contracture due to articular components such as bone, cartilage, synovium, subsynovium, capsule, and ligaments. These ROMs were calculated using the methods of Moriyama et al. [25]. The formulas are as follows:

$$ \begin{array}{l}\mathrm{Myogenic}\ \mathrm{restriction} = \mathrm{ROM}-\mathrm{m}\ \hbox{--}\ \mathrm{ROM}+\mathrm{m}\ \left(\mathrm{within}\ \mathrm{each}\ \mathrm{treatment}\ \mathrm{group}\right)\\ {}\mathrm{Arthrogenic}\ \mathrm{restriction} = \mathrm{ROM}-\mathrm{m}\ \mathrm{of}\ \mathrm{control}\ \mathrm{group}\ \hbox{--}\ \mathrm{ROM}-\mathrm{m}\ \mathrm{of}\ \mathrm{each}\ \mathrm{treatment}\ \mathrm{group}.\end{array} $$

### Tissue preparation

After the ROM measurement, the semitendinosus muscles attached to the bones (ischium and tibia), were immediately removed from both hindlimbs. The distal end of the muscles were fixed and the proximal end was connected to 16 g of weight to stretch them with a uniform force using a pulley in 0.1 M phosphate-buffered 4 % paraformaldehyde (pH 7.4) for 4 h at room temperature. The weight was determined beforehand as the same force required for the knee joint extension at 140 °, which is approximately the maximal extension ROM in a normal joint. After fixation, muscle length was measured using manually operated calipers (Mitutoyo Instruments, Yokohama, Tokyo) and the mid-belly portion of muscles was dissected and embedded in paraffin. Longitudinal sections (4-μm) were cut and stained with hematoxylin and eosin (HE) for microscopic observation.

Joint tissues were also dissected and immersion-fixed in the same fixative for 2 days at 4 °C to investigate knee joint pathology. During fixation, the knee joint angle was kept at 90 ° flexion. Joint samples were decalcified in 17.7 % ethylendiamintetraacetic acid (pH 7.2, OSTEOSOFT, Merck Millipore, Darmstadt, Germany) for 4 weeks at room temperature, cut into two pieces on the sagittal plane, and embedded in paraffin. The sections (4-μm) were cut at the central level of the medial condyle and stained with HE and aldehyde-fuchsin-Masson Goldner (AFMG) staining for histological study.

### Histological and histomorphometrical analysis

#### The semitendinosus muscles

Three portions from each HE section were photographed at a magnification of 20x with a digital camera mounted on a light microscope to visualize sarcomeres. Ten muscle fibers from each sample were randomly selected and the myofibril lengths were measured using Image J 1.48 software (National Institutes of Health, Bethesda, MD, USA). The number of serially arranged sarcomeres, including the length-measured myofibrils was counted and the average sarcomere lengths were calculated. In addition, the number of serially arranged sarcomeres, including the whole muscle, was calculated by dividing the muscle length by sarcomere length and expressing it as a total sarcomere number.

#### Knee joint hemorrhage score

Intra-articular hemorrhages were detected through microscopic observation in the knee joints in both Im and Im + CBX groups. Two observers unaware of the source of the samples evaluated joint hemorrhage severity. Severity of the hemorrhage was graded from 0 to 4 depending on the amount of infiltrated blood-derived cells: 0 = none, 1 = slight, 2 = moderate, 3 = severe, and 4 = more severe. The antero-superior, antero-inferior, postero-superior, and postero-inferior portions of joint cavities were graded separately. The sum of these individual scores averaged for the two observers was used as the representative score for each portions.

#### Joint capsule length

The length of the postero-superior and postero-inferior synovial membrane were measured using the image analysis software ImageJ according to the methods described by Ando et al. [26]. The measurements were summed to provide a total synovial membrane length. Shortened posterior synovial length is thought to be responsible for knee joint contracture in a rat immobilization and spinal cord injury model [25, 27, 28].

### Statistical analysis

All data were expressed as mean ± standard deviation (SD). Statistical analysis was performed using Dr. SPSS II for Windows (SPSS Japan Inc, Tokyo, Japan). For all data except for the hemorrhage score, one-way analysis of variance (ANOVA) and Tukey’s post-hoc test were applied. For the hemorrhage score, a non-parametric Kruskal-Wallis test was applied, followed by a Mann-Whitney test with the Bonferroni adjustment. A *P*-value of < 0.05 was considered statistically significant.

## Results

### Range of motion

**Fig. 2:**
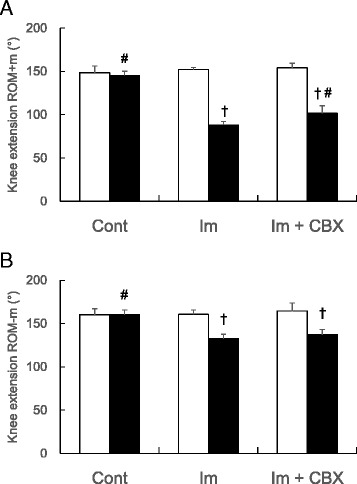
Histograms showing changes in passive knee extension ROMs. **a** ROM before myotomy of knee flexor muscles (ROM+m). Joint immobilization induced ROM+m restrictions in both Im and Im + CBX groups. Celecoxib partially prevented the ROM+m restriction induced by joint immobilization. **b** ROM after myotomy of knee flexor muscles (ROM-m). Joint immobilization induced ROM-m restrictions in both Im and Im + CBX groups. Celecoxib did not change ROM-m restriction induced by joint immobilization. (Experimental side (Rt), ■; Contralateral side (Lt), □). ^†^ *P* < 0.05 vs. contralateral side of the same group; ^#^ *P* < 0.05 vs. ipsilateral side of Im group. Data are expressed as means ± SD (*n* = 10)

Knee extension ROMs before myotomy (ROM+m) and after myotomy (ROM-m) of knee flexor muscles on both sides are shown in Fig. 2. The ROM+m on the experimental (Rt) side were 145 ± 5, 88 ± 4, and 102 ± 9 ° in the control, Im, and Im + CBX groups, respectively (Fig. 2a). The ROM+m on the experimental side decreased in both Im and Im + CBX groups compared to each contralateral (Lt) side and the control group (*P* < 0.0005, F = 240.9, η_P_^2^ = 0.95, one-way ANOVA; all *P* < 0.05, Tukey’s post hoc test). In comparing the two immobilized groups, ROM + m of the Im + CBX group was larger than the Im group (*P* < 0.05, Tukey’s post hoc test). The ROM-m on the experimental side were 160 ± 7, 132 ± 5, and 137 ± 9 ° in the control, Im, and Im + CBX groups, respectively (Fig. 2b). The ROM-m on the experimental side also decreased in both Im and Im + CBX groups compared to each contralateral side and the control group (*P* < 0.0005, F = 41.7, η_P_^2^ = 0.75, one-way ANOVA; all *P* < 0.05, Tukey’s post hoc test). Contrary to the result in ROM+m, there was no difference in ROM-m between the Im and Im + CBX groups (*P* = 0.38, Tukey’s post hoc test).

**Fig. 3:**
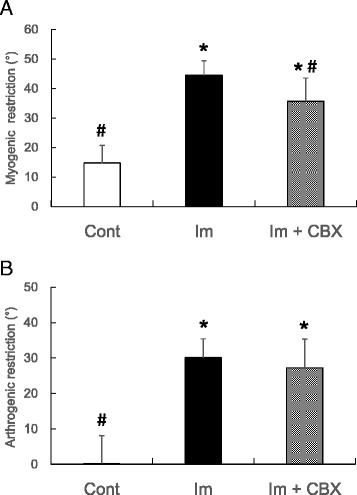
Histograms showing changes in knee extension ROM restriction. **a** Myogenic restrictions. Celecoxib treatment partially reduced myogenic contracture induced by joint immobilization. **b** Arthrogenic restriction. Celecoxib treatment did not reduce arthrogenic contracture induced by joint immobilization. *P < 0.05 vs. control group; ^#^ *P* < 0.05 vs. Im group. Data are expressed as means ± SD (*n* = 10)

Myogenic ROM restrictions were 15 ± 6, 44 ± 5 and 36 ± 8 ° in the control, Im, and Im + CBX groups, respectively (Fig. 3a). The myogenic ROM restrictions were significantly larger in both Im and Im + CBX groups compared to the control (*P* < 0.0005, F = 60.4, η_P_^2^ = 0.81, one-way ANOVA; both *P* < 0.05, Tukey’s post hoc test), and myogenic ROM restriction in the Im + CBX group was smaller than in the Im group (*P* < 0.05, Tukey’s post hoc test). Arthrogenic ROM restrictions were 0 ± 8, 30 ± 5, and 27 ± 8 ° in the control, Im, and Im + CBX groups, respectively (Fig. 3b). Arthrogenic ROM restrictions in the Im and Im + CBX groups were also significantly larger compared to the control (*P* < 0.0005, F = 54.3, η_P_^2^ = 0.80 for the ANOVA; both *P* < 0.05 for the Tukey’s post hoc test). There was no difference in arthrogenic ROM restriction between the Im and Im + CBX groups.

### Muscle length, sarcomere length, and serial number

**Fig. 4:**
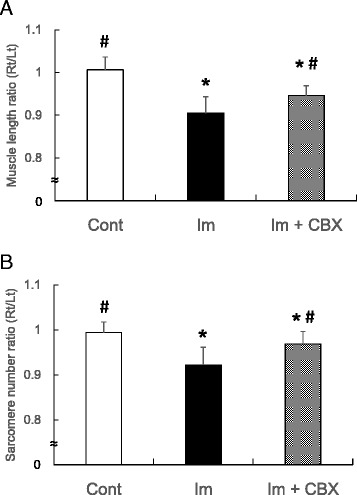
Histograms showing morphological changes in the semitendinosus muscles. **a** Muscle length ratio (experimental side (Rt)/contralateral side (Lt)). **b** Sarcomere number ratio (experimental side (Rt)/contralateral side (Lt)). Decrease in both muscle length ratio and sarcomere number ratio were partially prevented by celecoxib treatment. *P < 0.05 vs. control group; ^#^ *P* < 0.05 vs. Im group. Data are expressed as means ± SD (*n* = 10)

The semitendinosus muscle length, sarcomere length, and serial number are shown in Table 1. Muscle length on the experimental side decreased in both the Im and Im + CBX groups compared to each contralateral side and the control (*P* < 0.05). The muscle length ratios (experimental side/contralateral side) of the two immobilized groups were significantly decreased compared to the control. Between the two immobilized groups, the muscle length ratio of the Im + CBX group was larger than the Im group (*P* < 0.05, Fig. 4a). Changes in sarcomere number were similar to those in muscle length ratio (Fig. 4b), but there were no differences in sarcomere length among the three groups (Table 1).

**Table 1:** Semitendinosus muscle length, sarcomere length, and serial sarcomere number

Group		Muscle		Sarcomere			
		Length (mm)	Rt/Lt ratio (%)	Length (μm)	Rt/Lt ratio (%)	Number	Rt/Lt ratio (%)
Control	Rt	51.1 ± 1.3	101 ± 3	2.7 ± 0.2	101 ± 3	20016 ± 691	99 ± 2
	Lt	50.8 ± 1.5		2.7 ± 0.2		20165 ± 625	
Im	Rt	46.4 ± 2.1*	92 ± 5^†^	2.7 ± 0.1	98 ± 4	16892 ± 316*	92 ± 4^†^
	Lt	50.8 ± 0.6		2.8 ± 0.1		18047 ± 505	
Im + CBX	Rt	46.6 ± 0.9*	97 ± 3^†§^	2.8 ± 0.1	98 ± 4	16933 ± 316*	97 ± 3^†§^
	Lt	49.1 ± 0.8		2.8 ± 0.1		17291 ± 155	

Values are shown as means ± SD

*indicates significant difference from contralateral (Lt) side (*P* < 0.05)

^†^indicates significant difference from Control (*P* < 0.05)

^§^indicates significant difference from Im (*P* < 0.05)

### Joint histology and histomorphometry

In the semitendinosus muscles, muscle fibers were orderly arranged and apparent signs of inflammation were hardly observable in all three groups.

**Fig. 5:**
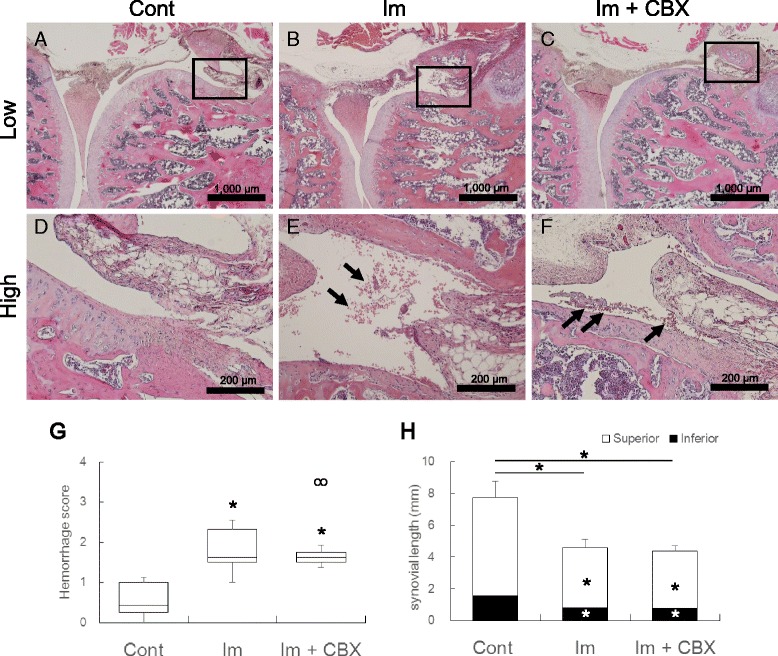
Morphological changes in the knee joint. Representative images of the posterior region of the knee with Hematoxylin and Eosin stain in the control group (**a**, **d**), Im group (**b**, **e**), and Im + BTX group (**c**, **f**). Higher magnification of the squared areas in **a**, **c**, and **e**, are shown in **b**, **d**, and **f**, respectively. In the control group, blood cell infiltration was hardly visible in the joint space at inferior capsule-cartilage junctions. In the Im and Im + CBX groups, comparable amounts of red blood cells were present (*arrows*). **g** Box plot showing mean values of intra-articular hemorrhage scores. Celecoxib treatment did not prevent joint hemorrhage induced by joint immobilization. ^*^ *P* < 0.05 compared with control. **h** Histogram showing posterior synovial lengths. Celecoxib treatment did not prevent the reduction of synovial length induced by joint immobilization. *P < 0.05 compared with control. Data are expressed as means ± SD (*n* = 10)

Representative images of the posterior capsule of the knee joint in HE sections are shown in Fig. 5a-f. In the control group, the joint capsule was thin and densely stained. Infiltrated cells were scarcely seen throughout the knee joint tissue (Fig. 5a, d). In AFMG sections, posterior joint capsules were densely stained green, indicating collagen fibers. In both Im and Im + CBX groups, the posterior capsules were thickened with edema and stained pale-green in AFMG sections. Fibrotic changes characterized by increased collagen deposition was not apparent in both Im and Im + CBX groups. Blood-derived cells (mainly red blood cells) had increased, especially in postero-superior portions near the synovial-cartilage junction in the knee joint cavities. Inflammatory infiltrates were rarely seen in posterior joint capsules (Table 2, Fig. 5b, c, e, f). These pathological changes were approximately comparable between Im and Im + CBX groups. Mean hemorrhage scores showed a significant increase in both Im and Im + CBX groups compared to the control (*P* < 0.05), but there was no difference between the Im and Im + CBX groups (Fig. 5g).

**Table 2:** Hemorrhage scores in each part of the knee joint cavity

Group	Antero-		Postero-		Average
	Superior	Inferior	Superior	Inferior	
Control	0.5 ± 0.5	0.5 ± 0.5	0.6 ± 0.5	0.5 ± 0.5	0.5 ± 0.4
Im	1.8 ± 0.8^†^	1.6 ± 0.5^†^	2.3 ± 1.1^†^	1.4 ± 1.0^†^	1.8 ± 0.6^†^
Im + CBX	1.5 ± 0.8^†^	1.3 ± 0.8^†^	2.0 ± 0.8^†^	1.7 ± 1.1^†^	1.8 ± 0.6^†^

Values are shown as means ± SD

^†^ indicates significant difference from Control (*P* < 0.05)

The postero-superior synovial lengths were 6.2 ± 1.0 mm, 3.8 ± 0.5 mm (*P* < 0.05), and 3.61 ± 0.3 mm (*P* < 0.05) in the control, Im, and Im + CBX groups, respectively. The postero-inferior synovial lengths were 1.6 ± 0.2 mm, 0.8 ± 0.2 mm (*P* < 0.05), and 0.8 ± 0.3 mm (*P* < 0.05), and the total lengths of the posterior synovial membrane were 7.7 ± 1.1 mm, 4.6 ± 0.5 mm (*P* < 0.05), and 4.4 ± 0.3 mm (*P* < 0.05) in the control, Im, and Im + CBX groups, respectively. The posterior synovial membranes in the Im and Im + CBX groups were significantly shorter compared to the control (Fig. 5h). However, there was no difference between the two immobilized groups in any of the synovial length parameters.

## Discussion

The key finding of the present study is that the COX-2 inhibitor celecoxib can partially prevent myogenic contracture by acting on knee flexor muscles following joint immobilization. However, arthrogenic contracture could not be inhibited by celecoxib treatment due to changes in articular components. These results indicate that the COX-2 mediated pathway, which is involved in inflammation and nociception, contributes to the formation of myogenic contracture independently of joint immobilization. This study is therefore the first to provide the beneficial effects of NSAID on the prevention of immobilization-induced joint contracture.

The passive extensibility of skeletal muscle is determined by several anatomic and physiologic properties, with maximal muscle length being one important determinant of joint ROM [1, 29]. In this study, we demonstrated that the length of semitendinosus muscles in immobilized knee joints shortened resulting from a reduction in sarcomere number. This is in accordance with previous reports [29]. These changes were attenuated by celecoxib treatment concomitantly with myogenic ROM restriction. These results suggest that the COX-2 mediated pathway is involved in the regulation of muscle length by changing sarcomere number in the immobilized joint. The COX-2 also regulates skeletal muscle regeneration [30, 31], hypertrophy [32], and recovery from atrophy [33]. Passive extensibility is influenced by muscle size [29]. However, the effects of COX-2 inhibitor on muscle atrophy following immobilization is unknown. Further study on muscle atrophy may elucidate the mechanisms behind CBX-mediated attenuation of muscular contracture.

In guinea pigs, muscle contraction in a shortened position accelerates the reduction in extensibility with sarcomere loss [34]. Huet de la Tour et al. [35] also reported that spasm of soleus muscles elicit a decrease in passive extensibility and a marked reduction of sarcomere number in guinea pigs. These results suggests that muscle contraction in a shortened position is key in the determination of muscle length. In addition, knee joint nociception also induces involuntary muscle contraction in biceps femoris muscles [36]. Several reports indicate that joint immobilization induces inflammatory conditions within affected joints, such as an increase in joint breadth [19], synovial cell proliferation, and mononuclear infiltration [13], as well as an upregulation of inflammatory cytokines [14]. In addition, we found pathological changes such as blood cell infiltration into intracapsular spaces and joint capsule edema in Im and Im + CBX groups. Interestingly, it has been reported that COX-1 and COX-2 are upregulated in synoviocytes and chondrocytes in immobilized rat knee joints [37]. Celecoxib inhibits prostaglandin E_2_ synthase, mediated by COX-2, and relieves pain [23]. Taken together, successful celecoxib treatment for alleviating myogenic contracture may be due to inhibition of the reduction in sarcomere number and muscle length via inhibition of the COX-2-mediated inflammatory nociception following joint immobilization.

In addition, qualitative and quantitative changes in connective tissue are believed to be a leading candidate as the cause of myogenic contracture [1, 29]. Endo- and perimysial connective tissue have been shown to increase in the rat soleus muscle when immobilized for 3 weeks [38, 39]. Perimysial collagen fibril arrangement also alters in immobilized rat soleus muscles [38] with a concomitant reduction in joint ROM [3]. In the present study, the sarcomere length at a stretched position, which is theoretically inhibited by the connective tissue surrounding the muscle fibers and bundles, was unaltered by joint immobilization. However, this result does not necessarily deny the involvement of intramuscular connective tissue in muscle extensibility. The semitendinosus muscles were pulled by a weight of 16 g, and a stronger or weaker traction was not tested. In addition, changes in mechanical properties may occur in non-semitendinosus muscle, or in sliding between adjacent muscles. Thus, further study is needed to determine the full effects of celecoxib on the volume and mechanical properties of intramuscular collagen.

Celecoxib had no effect on arthrogenic contracture, unlike myogenic contracture. Trudel et al. [4] noted that structural reorganization in the joint posterior capsule (shortening due to adhesion between folds, type I collagen deposition, and advanced glycation end products) is responsible for arthrogenic joint contracture following long-lasting (>4 weeks) joint immobilization in flexion [4]. In this study, the lengths of the posterior capsules were comparably shortened in both Im and Im + CBX groups. Celecoxib had no effect on arthrogenic contracture formation, which may be due to a failure to inhibit the shortening of the joint capsule. This result also suggests that the joint capsule length may be shortened by joint immobilization regardless of the inflammation.

Several studies report that the prevention of arthrogenic contracture is achieved by intraarticular injection of decorin, which inhibits the activity of transforming growth factor-beta [40], hyaluronic acid [21, 22], mytomycin C [41], and celecoxib [42]. These previous reports used animal models of traumatic contracture or a combination of joint trauma and immobilization, both of which show prominent inflammatory changes in affected joints, unlike in an immobilized model. In an immobilized model, arthrogenic contracture is alleviated by intra-articular injection of corticosteroid triamcinolone in rats [43]. However, as an adverse effect, triamcinolone-treated rat joints showed a disruption with a significantly lighter weight load than the untreated control. A recent study showed that intra-articular injection of hyaluronan could prevent fibrotic and inflammatory changes in immobilized joint capsules [21]. Exogenous hyaluronan treatment stimulates hyaluronan synthesis and reduces pro-inflammatory mediators in osteoarthritic joints [44]. The reason for this discrepancy between celecoxib and hyaluronan remain unexplained. Actions except anti-inflammation of hyaluronan, e.g., hydrodynamic changes, might be in part due to inhibit arthrogenic contracture.

Interestingly, microscopic observation revealed that intra-articular hemorrhages increased comparably in both Im and Im + CBX groups compared to the control. To test the possibility of developing joint damage caused by the mechanical stress during ROM measurement, we performed histological observation in three-week-immobilized knee joints without ROM measurement, additively (*n* = 2). The observation confirmed that joint immobilization itself contributes to bleeding. Coagulating blood not only induces joint damage [45] but also accelerates immobilization-induced synovial adhesions and capsular shortening in rats [28]. The mechanism of joint hemorrhage induced by joint immobilization is unknown. However, if it is possible to prevent joint hemorrhage during immobilization, arthrogenic joint contracture might be alleviated.

Generally, passive movement is used as a strategy for the treatment of joint contracture. However, joint movement cannot be done in acute post-surgery phases or for orthopedic diseases such as joint fractures, muscle, and ligament injuries. In addition, it is not clear whether these interventions are clinically effective on joint mobility [9, 10]. An important finding of this study is that even immobilization-induced contracture can be attenuated by oral administration of celecoxib, which is advantageous because it can be provided during periods of immobilization. This suggests that anti-inflammatory drug celecoxib is a possible option for the prevention of joint contracture followed by immobilization.

There are several limitations to the study. First, we observed the effects of joint immobilization and celecoxib treatment on joint contracture formation at only one time point. However, changes in the degree of contribution of both articular and muscle components in joint contracture formation depend on the period of immobilization [4]. Second, we measured the ROMs with only a single load, not an escalating load which provide a more detailed analysis of immobility. Third, administration of celecoxib in this study (50 mg/kg dose) is approximately 10X higher than that used by humans. In addition, the half-life for celecoxib in male rats is significantly shorter than that in human (3.7 h vs 11 h) [46]. Fourth, COX-2 activity was not measured to confirm drug efficacy and we did not investigate the roles of COX-2 pathways on the formation of joint contracture. In addition, we have not directly shown the anti-inflammatory effects of Celecoxib in immobilized joints. Therefore, whether this celecoxib efficacy could be extended for clinical use of joint contracture still requires further studies.

## Conclusions

The present study revealed that the oral administration of COX-2 inhibitor celecoxib partially reduced myogenic ROM restriction concomitantly with knee flexor muscle shortening following immobilization. This result implies that inflammation and nociception are involved in myogenic contracture formation independently of joint immobilization. Celecoxib had a significant efficacy for prevention of joint contracture following immobilization in a rat model.

## Abbreviations

CBX: Celecoxib
COX: Cyclooxygenase
HE: Hematoxylin and eosin
Im: Immobilization
NSAID: Non-steroidal anti-inflammatory drug
ROM: Range of motion
